# Identification of Transient Receptor Potential Vanilloid 3 Antagonists from *Achillea alpina* L. and Separation by Liquid-Liquid-Refining Extraction and High-Speed Counter-Current Chromatography

**DOI:** 10.3390/molecules25092025

**Published:** 2020-04-26

**Authors:** Shi-Wei Sun, Rong-Rong Wang, Xiao-Ying Sun, Jia-He Fan, Hang Qi, Yang Liu, Guo-Qing Qin, Wei Wang

**Affiliations:** Department of Natural Medicine and Pharmacognosy, School of Pharmacy, Qingdao University, Qingdao 266071, China

**Keywords:** *Achillea alpina*, isochlorogenic acid, transient receptor potential vanilloid 3 channel, high-speed counter-current chromatography, molecular docking, liquid-liquid extraction

## Abstract

Bioassay-guided fractionation of the ethanol extract of whole herbs of *Achillea* *alpina* led to the isolation of isochlorogenic acids A and B as transient receptor potential vanilloid 3 (TRPV3) channel antagonists by using a calcium fluorescent assay. The structures were identified by spectroscopic analysis and the inhibitory activities of isochlorogenic acids A and B were confirmed by whole-cell patch clamp recordings of human embryonic kidney 293 (HEK293) cells expressing human TRPV3. Molecular docking results revealed that these two compounds reside in the same active pocket of human TRPV3 channel protein with lower binding energy than the agonist 2-aminoethoxydiphenyl borate (2-APB). High-speed counter-current chromatography (HSCCC) coupled with a liquid-liquid extraction approach was successfully established for the separation of isochlorogenic acids A and B from the whole herbs of *A*. *alpina*. Ethyl acetate and *n*-hexane-ethyl acetate-water (3:3:4 and 1:5:4, v/v/v) were selected as liquid-liquid extraction solvent systems to remove high- and low-polarity impurities in the mixture. Sixty g of ethanol extract was refined by solvent partition to yield 1.7 g of the enriched fraction, of which 480 mg in turn obtained 52.5 mg of isochlorogenic acid B (purity 98.3%) and 37.6 mg isochlorogenic acid A (purity 96.2%) after HSCCC with *n*-hexane-ethyl acetate-water containing 1% acetic acid (1:4:8, v/v/v).

## 1. Introduction

Thermal transient receptor potential (TRP) channels, known as multimode sensors, play crucial functions in numerous physiological and pathophysiological processes by integrating a variety of physical and chemical stimuli in cellular signaling [1]. Transient receptor potential vanilloid 3 (TRPV3), a member of the vanilloid TRP subfamily, is expressed abundantly in the epidermal keratinocytes and is involved in the skin barrier formation, hair growth, cutaneous growth and survival, and temperature sensation [2]. TRPV3 channel dysfunction caused by genetic gain-of-function mutations or pharmaceutical activations is associated with numerous human skin diseases including skin inflammation, cutaneous pain, chronic itch, atopic dermatitis, and Olmsted syndrome [3,4]. All these observations strongly suggest that TRPV3 antagonists might be useful tools in the treatment and management of various skin diseases [5]. Phytochemicals have attracted attention as source materials for the development of new drug or alternative therapy for the management of diseases. However, few natural compounds have been described to inhibit TRPV3 [6,7,8]. Thus, the search for natural TRPV3 inhibitors for the remedy of skin diseases continues unabated.

*Achillea alpina* L. is a perennial herbaceous plant belonging to Asteraceae, mainly distributed in southern China and eastern Asia. As a horticulture crop it has not only ornamental value but also good materials for fresh cut flowers. As a traditional Chinese medicine, the aerial parts of *A*. *alpina* were recorded in Pharmacopoeia of the People’s Republic of China with effects of detoxification, clearing dampness, blood circulation promotion, and relieving pain. In terms of phytochemistry, studies of the extracts of the aerial parts of *A*. *alpina* led to the isolation of organic acids, flavonoids, terpenoids, alkaloids, steroids, lignanoids, quinones, and several other compounds, some of which exhibited antioxidant, antimelanogenic, anti-inflammatory, antipyretic, analgesic, sedative, and cardiovascular protective activities [9,10,11,12,13,14]. In our continuing search for TRPV3 channel antagonists from medicinal plants [7,8], the ethanol extract of whole herbs of *A*. *alpina* showed an inhibitory activity on TRPV3 channel by using a calcium fluorescent assay. Subsequent bioassay-guided investigation led to the isolation of isochlorogenic acids A and B as TRPV3 channel antagonists. In view of their TRPV3 channel antagonist effects, the separation of sufficient amounts of isochlorogenic acids A and B is urgently needed to provide the foundation for further application investigations. However, the traditional column chromatography separation methods that we used to discover bioactive compounds have many disadvantages, such as repeated column separation, which lead to time consumption and lower recovery. Thus, to meet the demand, developing a rapid and effective separation method is critical. High-speed counter-current chromatography (HSCCC) is a solid support-free liquid-liquid partitioning chromatography with the advantages of saving operation time and avoiding low yield, which has recently been applied for the separation and purification of the bioactive molecules from natural products. Although the HSCCC methods for separation of isochlorogenic acid derivatives from *Lonicera japonica* have been reported in the previous studies [15,16,17], they cannot be directly applied to the separation of isochlorogenic acids A and B from the whole herbs of *A*. *alpina* due to the interruption by the impurities in complex mixture. In order to explore the crop resources of *A*. *alpina*, a comprehensive separation method was developed by HSCCC coupled with a liquid-liquid extraction strategy in this research. The two-phase solvent systems for sample liquid-liquid extraction pretreatment and HSCCC separation with a modification of the polarity and acidity were simultaneously established.

## 2. Results and Discussion

### 2.1. Bioassay-Guided Fractionation and Structure Identification

**Figure 1:**
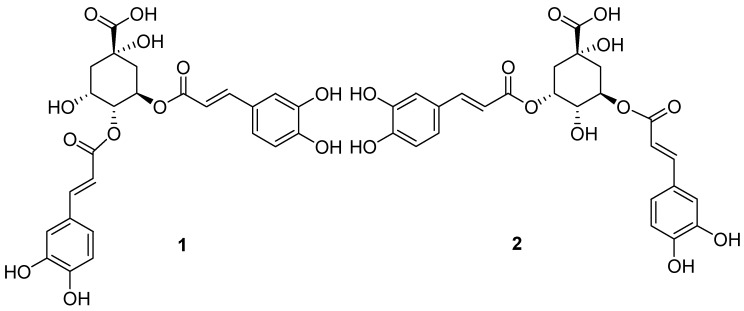
Structures of isochlorogenic acids B (**1**) and A (**2**).

Bioassay-guided fractionation of the ethanol extract of the dried whole herbs of *A*. *alpina* was performed to search for the bioactive constituents responsible for the inhibitory activity on TRPV3 channel. Liquid-liquid extraction is the simplest and most effective method for the separation of complex mixtures, which is widely used in the first step of extract separation [18,19]. So, the extract was initially partitioned by sequential solvent extraction with *n*-hexane, ethyl acetate, and *n*-butanol. The pharmacological results showed that the ethyl acetate fraction exhibited significant TRPV3 channel inhibitory activity. Nylon-6, a polyamide resin, has found interest in the chromatographic separation of bioactive substances due to its unique mechanism and easy regeneration [20,21]. The active fraction was chromatographed on a polyamide resin column, and seven subfractions were collected, among which the subfraction eluted by 50% ethanol in water (v/v) was the most active. Following the second Sephadex LH-20 column, because they have the same molecular weight and only differ in the position of one caffeoyl group, it was difficult to obtain the individual compounds. Two compounds were isolated with further separation of the subfraction by semi-preparative HPLC, and their structures were characterized as isochlorogenic acid B (**1**) and isochlorogenic acid A (**2**) using NMR and MS spectra, as shown in Figure 1.

### 2.2. Identification of Isochlorogenic Acids A and B as TRPV3 Channel Antagonists

**Figure 2:**
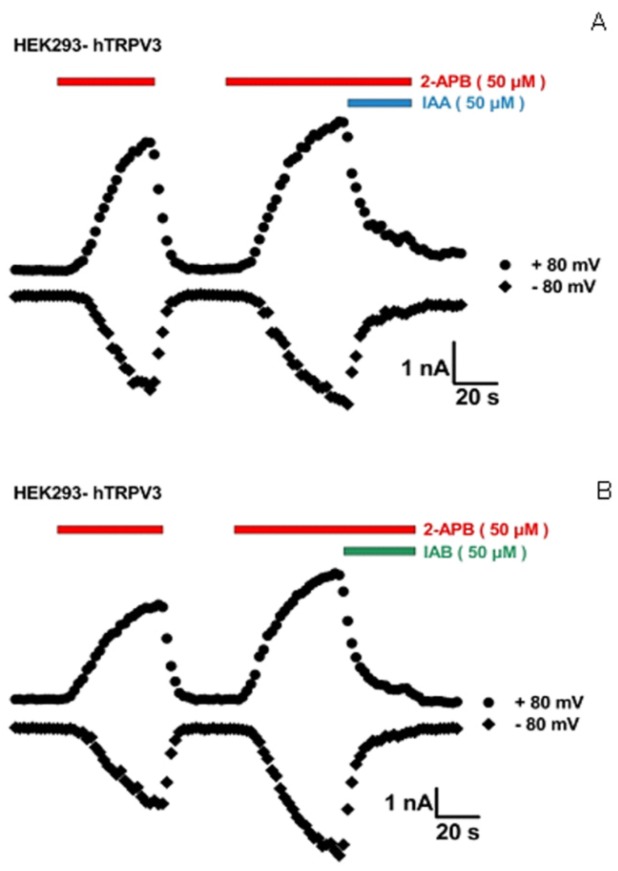
Isochlorogenic acids A and B inhibit TRPV3-mediated current activated by 2-APB. (**A**) Representative hTRPV3 current activated by 50 μM 2-APB (red bar) before and after its coapplication with 50 μM isochlorogenic acid A (blue bar) and washout. (**B**) Representative hTRPV3 current activated by 50 μM 2-APB (red bar) before and after its coapplication with 50 μM isochlorogenic acid B (green bar) and washout.

The patch-clamp technique, an advanced electrophysiological technique, has been widely used for ion channel research as a ‘gold standard’. The inhibitory activities of isochlorogenic acids A and B on TRPV3 channel were confirmed using whole-cell patch-clamp recordings of human embryonic kidney 293 (HEK293) cells expressing TRPV3 channels. As shown in Figure 2, 50 μM of isochlorogenic acids A and B can significantly reduce TRPV3 current activated by 50 μM 2-aminoethoxydiphenyl borate (2-APB) with the inhibition rates of 81.8 ± 2.6% (n = 8) and 90.6 ± 2.7% (n = 7), respectively.

Isochlorogenic acids A and B are region isomers of each other, both of which are well-known caffeoylquinic acid derivatives in the plant kingdom. However, this is the first report on the isolation of isochlorogenic acids A and B from the dried whole herbs of *A*. *alpina*. Accumulating evidences suggest that caffeoylquinic acid derivatives display a wide range of medicinal properties. For example, several studies have demonstrated that isochlorogenic acid A, a most common derivative of caffeoylquinic acids, has anti-inflammatory activity, which include prevention of lipopolysaccharide-induced injury in endothelial cells; inhibition of macrophages’ accumulation, reactive oxygen species, and nitric oxide productions, and interleukin-1*β* and tumor necrosis factor levels in an *in vivo* zebrafish model of cupric sulfate-induced and lipopolysaccharide-stimulated inflammation; decrease of nod-like receptor protein 3 (NLRP3) inflammatory complex activation and nuclear factor-kappa B phosphorylation in rats with collagen-induced arthritis; and so forth [22,23,24]. However, to the best of our knowledge, none of isochlorogenic acids A and B has been tested for action on TRPV3 channel. Previous studies have shown that stimulation of TRPV3 channel can induce a strong pro-inflammatory response in human epidermal keratinocytes [25]. Our results showed that isochlorogenic acids A and B are TRPV3 channel antagonists, which can provide a mechanistic explanation for their anti-inflammatory activities. However, there is a lack of in vivo studies on the inhibitory activities of isochlorogenic acids A and B on TRPV3 channel. Further investigations are required to use these compounds for anti-inflammatory therapy.

### 2.3. Molecular Docking Analysis

**Figure 3:**
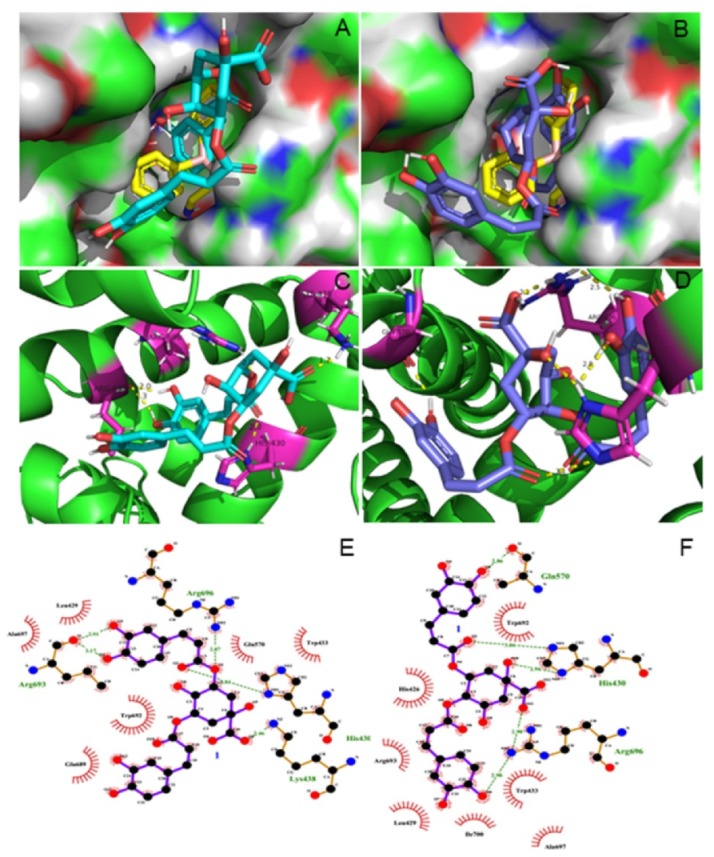
Docked conformations of isochlorogenic acid (**A**), isochlorogenic acid (**B**), and agonist 2-APB with cyan, slate, and yellow sticks into the active site of 6MHO protein, respectively (**A**,**B**). Analysis of molecular docking showing the key interactions in the binding pocket. The key residues are presented in the form of purple sticks, and hydrogen bonds are drawn with dotted yellow (**C**,**D**). Ligplot^+^ of isochlorogenic acids A and B bound to 6MHO protein showing the key hydrophobic interactions (**E**,**F**).

The structures of apo and sensitized human transient receptor potential vanilloid 3 (hTRPV3) were presented recently, as well as several structures of TRPV3 in the presence of the common thermos TRPV agonist 2-APB [26]. We tried to explore the potential binding sites for isochlorogenic acids A and B using the AutoDock 4.2 program based on the presented structures [27]. The two isolated compounds were docked into the hTRPV3 protein and found that these two compounds reside in the same active pocket as the agonist 2-APB, as a result of the resemblance of chemical structures between the ligands and agonist 2-APB (Figure 3). The 2-APB binding site, which was identified in the domain between linker and TRP-Box, possessed two key residues (His426 and Arg696) specifically required for sensitivity for TRPV3 to 2-APB [28,29].

As shown in Figure 3, isochlorogenic acids A and B bound very well at the 2-APB binding site with the lower binding energy of −6.55 and −6.85 kcal/mol, respectively, while 2-APB was −5.43 kcal/mol. The docking result of isochlorogenic acid A showed that five H-bonds were formed with four key residues including His430, Lys438, Arg693, and Arg696. The 1-carboxy group was able to hydrogen bond with Lys438, while 9″-carbaryl group participated in hydrogen bonding with His430 and the oxygen atom that was adjacent to 9″-carbanyl oriented toward Arg696. In addition, 3″- and 4″-hydroxyl group made H-bonds with Arg693. Furthermore, there were hydrophobic interactions with Leu429, Trp433, Gln570, Glu689, Trp692, and Ala697. The docking result of isochlorogenic acid B indicated that five hydrogen bonds were formed with three key residues including His430, Gln570, and Arg696. The 1-hydroxyl group and 9″-carbanyl group in isochlorogenic acid B were able to hydrogen bond with His430, while 1-carboxy group and 4′-hydroxyl group as the hydrogen-bond partner oriented toward Arg696. Additionally, 4″-hydroxyl group participated in hydrogen bonding with Gln570. Besides, isochlorogenic acid B had hydrophobic interaction with His426, Leu429, Trp433, Trp692, Arg693, Ala697, and Ile700. The result indicated that isochlorogenic acids A and B occupied the same pocket of 2-APB, owing to interaction with the same key residues His430 and Arg696, which are important to mediate the open or closed state of TRPV3 receptor. However, this putative binding site by molecular docking of isochlorogenic acids A and B into hTRPV3 receptor needs to be further verified by site-specific mutagenesis or other molecular biological techniques.

In recent years, molecular docking has been becoming a useful approach in analysis of TRPV3 to enrich our understanding of the active compounds on the inhibition effect of TRPV3 channel function [8,30]. For example, the molecular docking program was performed to identify the potential TRPV3 binding sites for forsythoside B, and the docking results showed forsythoside B inhibited TRPV3 by binding to the region between the selectivity filter and the central cavity of TRPV3 [8].

### 2.4. HPLC Analysis

To develop an HPLC method for the sample analysis, the target compounds were firstly affirmed by comparing with the reference substances of isochlorogenic acids A and B on the HPLC chromatogram. Several solvent systems, such as acetonitrile-water, acetonitrile-water containing 2% acetic acid, methanol-water, and methanol-water containing 2% acetic acid with different gradient elution mode, were employed and optimized. The results showed that adding acetic acid into the mobile phase can alleviate the peak tailing of compositions in the sample. The optimum HPLC analysis conditions required a proper separation between the peaks of isochlorogenic acids A and B and other peaks. Good separation conditions can be acquired when acetonitrile-water containing 2% acetic acid was used as the mobile phase in gradient elution mode as follows: Acetonitrile: 0–10 min, 6%–6%; 10–20 min, 6%–10%; 20–45 min, 10%–20%; 45–80 min, 20%–35% at a flow rate of 1.0 mL/min.

### 2.5. Selection of Extraction Solvent Systems for Sample Pretreatment

**Figure 4:**
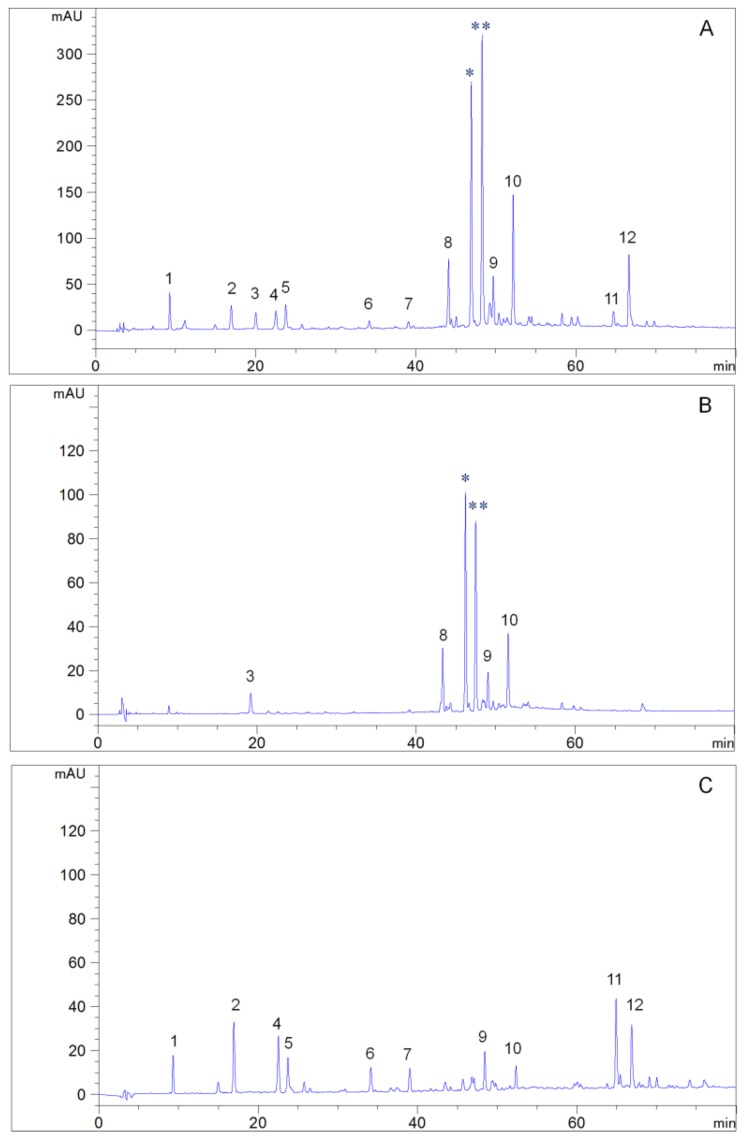
HPLC chromatograms of the crude sample and isolated fractions. Column: YMC-Pack ODS-A (250 × 4.6 mm, 5µm); mobile phase: Water containing 2% acetic acid as A and acetonitrile as B (B: 0–10 min, 6%–6%; 10–20 min, 6%–10%; 20–45 min, 10%–20%; 45–80 min, 20%–35%); flow rate: 1.0 mL/min; column temperature: 35 °C; detection wavelength: 254 nm. (**A**) Crude sample; (**B**) Fr. III; (**C**) Fr. II. Peaks: 1–12, impurities; * isochlorogenic acid B; ** isochlorogenic acid A.

Although the selection of a suitable two-phase solvent for HSCCC separation plays a critical role in obtaining the target compounds, the solvent system used in sample pretreatment needs to be paid attention to, reducing the nontarget impurities in the mixture, which will reduce the retention of stationary phase resulting in separation failure, interrupt separation resulting in the decrease of product purity, and increase operation time resulting in the decrease of yield. In the course of discovering bioactive compounds, the ethanol extract was suspended in water and partitioned with single water-immiscible organic solvents including *n*-hexane, ethyl acetate, and *n*-butanol. The chromatographic results showed that, in addition to the target compounds, a large number of impurities were found in the ethyl acetate fraction due to the large difference in polarity and poor selectivity of a single solvent. Recently, several multicomponent liquid-liquid extraction-assisted sample pretreatment systems have been established, which can effectively remove impurities. Moreover, without changing the components, the two-phase solvent systems can be used for HSCCC separation with a slight modification of the polarity [31,32]. In the present study, firstly, the ethyl acetate extract was dissolved and extracted with the lower phase and upper phase of n-hexane-ethyl acetate-water (3:3:4, v/v/v), respectively, to remove the nontarget impurities in the mixture. Then, the evaporated residue of the lower phase of *n*-hexane-ethyl acetate-water (3:3:4, v/v/v) was dissolved in the upper phase of *n*-hexane-ethyl acetate-water (1:5:4, v/v/v), and the sample rich in target compounds was obtained by partition with the upper phase of *n*-hexane-ethyl acetate-water (1:5:4, v/v/v). As can be seen in Figure 4, the target compounds, isochlorogenic acids A and B, along with the nontarget impurities 3 and 8–10 were found in fraction (Fr.) III, the nontarget impurities 1, 2, 4–7, and 9–12 were observed in Fr. II, and the mass of Fr. I was very small. According to the above results, *n*-hexane-ethyl acetate-water at the ratios of 3:3:4 and 1:5:4 (v/v/v) were used to extract the ethyl acetate extract in turn.

### 2.6. Selection of Two-Phase Solvent System for HSCCC

A successful separation of HSCCC depends on a suitable solvent system based on the ideal partition coefficients (*K*) of the target compounds, with a range of 0.2–5 [33]. In addition, the separation factor between the two compounds (α = K_1_/K_2_, K_1_ > K_2_) should be greater than 1.5 [34]. According to the results from the selection of solvent system for sample pretreatment based on liquid-liquid micro-extraction, if *n*-hexane-ethyl acetate-water at the ratios of 3:3:4 (v/v/v) as the solvent system was directly used for HSCCC separation, the target compounds would be eluted near the solvent front leading to difficulty in separation. On the contrary, the separation time with *n*-hexane-ethyl acetate-water at the ratios of 1:5:4 (v/v/v) as the solvent system would be very long. So, a series of two-phase solvent systems, *n*-hexane-ethyl acetate-water with different ratios, were further investigated and the *K* values of isochlorogenic acids A and B and the major nontarget impurity 9 were measured, and summarized in Table 1. Firstly, *n*-hexane-ethyl acetate-water at the ratios of 1:3:4 (v/v/v) was selected to test the distribution of three compounds and it was found that three compounds mainly distribute in the aqueous phase. Then, the ratios of ethyl acetate and water were changed to acquire suitable *K* values. The system *n*-hexane-ethyl acetate-water 1:4:8 (v/v/v) gave suitable *K* values for three compounds, but the *α* value between isochlorogenic acids A and the impurity 9 was unsuitable for satisfactory separation. By adding 1% acetic acid to the water, good *α* values were achieved between isochlorogenic acids A and B and the impurity 9, and the *K* values of three compounds were also suitable.

### 2.7. HSCCC Separation

**Figure 5:**
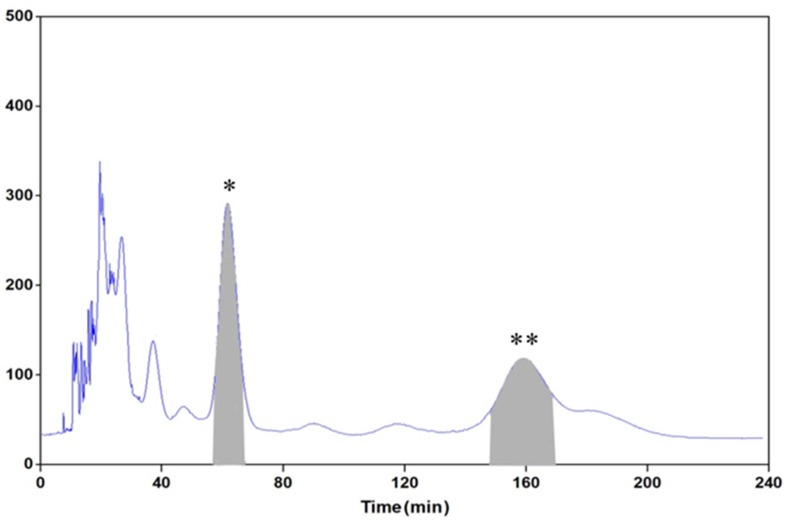
High-speed counter-current chromatography (HSCCC) chromatogram of the Fr. III. Experimental conditions: Two-phase solvent system: *n*-hexane-ethyl acetate-water containing 1% acetic acid (1:4:8, v/v/v); stationary phase: Upper phase; mobile phase: Lower phase; elution mode: Head to tail; flow rate: 3.0 mL/min; rotation speed: 900 rpm; separation temperature: 25 °C; detection wavelength: 254 nm; sample loading: 80 mg of the Fr. III dissolved in 5 mL of the lower phase; retention of the stationary phase: 71%. Peaks: *, isochlorogenic acid B; **, isochlorogenic acid A.

Under the rotation speed of 900 rpm, HSCCC separation was performed using the two-phase solvent system composed of *n*-hexane-ethyl acetate-water containing 1% acetic acid (1:4:8, v/v/v) at a flow rate of 3.0 mL/min (Figure 5). The aqueous phase was used as mobile phase, and the retention of stationary phase was 71%. After six successive separation operations, 52.5 mg of isochlorogenic acid B with the purity of 98.3% and 37.6 mg isochlorogenic acid A with purity of 96.2% were isolated from 480 mg of Fr. III.

## 3. Materials and Methods

### 3.1. Apparatus and Reagents

Semi-preparative HPLC equipment (Agilent technologies, Santa Clara, CA, USA) includes an Agilent PrepStar SD-1 pump, a ProStar UV-Vis detector, and a Shim Pak ODS column (250 × 21.2 mm, i.d., 10 μm, Shimadzu, Kyoto, Japan). Analytical HPLC was conducted on Agilent 1260 system (Agilent technologies, Santa Clara, CA, USA) equipped with a G1311C quaternary pump, a G1314F variable wavelength detector, a G1329B autosampler, and a YMC-Pack ODS-A column (250 × 4.6 mm, i.d., 5 μm, YMC, Kyoto, Japan). The HSCCC equipment (Tauto Biotechnique, Shanghai, China) was TBE-300C system with a TBP-5002 constant flow pump, three polytetrafluoroethylene preparative coil separation columns (i.d. of the tubing = 2.6 mm, total volume = 310 mL), a TBD-2000 ultraviolet detector, a DC-0506 constant temperature regulator, a 20 mL sample loop (Tauto Biotechnique, Shanghai, China), and an Easyobrun-1000 workstation (Sanotac Scientific Instruments, Shanghai, China).

HPLC-grade acetonitrile and methanol were purchased from Oceanpak (Goteborg, Sweden). All analytical grade organic solvents were bought from FuYu Fine Chemical Reagent (TianJin, China). Polyamide resin was obtained from Cangzhou Bon Adsorber Technology (Cangzhou, China).

### 3.2. Plant Materials

The dried whole herbs of *A*. *alpina* were bought from Yonggang Pharmaceutical Company, Bozhou, Anhui Province of China, in October 2016, and authenticated by Prof. Yingxia Li, School of Pharmacy, Qingdao University, China. A voucher specimen (accession number: AaSC20161001) was deposited at School of Pharmacy, Qingdao University, China.

### 3.3. Isolation and Identification

The dried whole herbs of *A*. *alpina* (2 kg) were cut into pieces and extracted three times at 50 °C with ethanol for 2 h. The obtained filtrates were combined and evaporated under vacuum at a temperature of 50 °C to obtain a residue (108 g). The residue was suspended in water and successively partitioned with *n*-hexane, ethyl acetate, and *n*-butanol, yielding *n*-hexane (24.1 g), ethyl acetate (5.9 g), and *n*-butanol (28.3 g) fractions, respectively. Among all obtained fractions, the ethyl acetate fraction showed good inhibition on TRPV3 channel through a calcium fluorescent assay. The ethyl acetate fraction was pulped with an equal amount of polyamide resin, dried, powdered, and subjected to column chromatography on polyamide resin, which was eluted initially with water, then subjected to gradient elution with different concentrations of ethanol in water (10%, 30%, 50%, 70%, and 90%, v/v) and finally with acetone. Seven eluted subfractions were obtained and their inhibitory activity was tested as described above. The subfraction eluted by 50% ethanol in water (v/v), which showed the most active subfraction on TRPV3 channel inhibitory bioassay, was purified by size exclusion chromatography using Sephadex LH-20 with an isocratic gradient of methanol followed by reversed phase HPLC using mobile phase 26% ethanol in water (v/v) to obtain compounds **1** (13.4 mg) and **2** (8.4 mg). 

The identification of isolated bioactive compounds was performed with NMR and ESI-MS spectra, which were recorded on a Bruker AV-500 FT-NMR spectrometer and a Bruker microTOFQ mass spectrometer, respectively (Bruker Daltonics, Bremen, Germany).

Isochlorogenic acid B (**1**): light yellow powder; ^1^H-NMR (500 MHz, CD_3_OD) *δ* 7.58 (1H, d, *J* = 15.9 Hz, H-7′), 7.50 (1H, d, *J* = 15.9 Hz, H-7″), 7.06 (1H, d, *J* = 2.0 Hz, H-2′), 7.01 (1H, d, *J* = 2.0 HZ, H-2″), 6.93 (1H, dd, *J* = 8.2, 2.0 Hz, H-6′), 6.88 (1H, dd, *J* = 8.2, 2.0 Hz, H-6″), 6.77 (1H, d, *J* = 8.2 HZ, H-5′), 6.74 (1H, d, *J* = 8.2 Hz, H-5″), 6.30 (1H, d, *J* = 15.9 Hz, H-8′), 6.24 (1H, d, *J* = 15.9 Hz, H-8″), 5.63 (1H, m, H-3), 5.19 (1H, m, H-4), 4.13 (1H, m, H-5), 2.18 (1H, dd, *J* = 13.5, 4.0 Hz, H-6a), 2.08 (1H, m, H-6b), 2.06 (2H, m, H-2); ^13^C-NMR (125 MHz, CD_3_OD) *δ* 182.0 (C-7), 168.4 (C-9′, 9″), 149.8 (C-4′), 149.6 (C-4″), 147.4 (C-7′), 147.2 (C-7″), 146.9 (C-3′), 146.8 (C-3″), 127.8 (C-1′), 127.7 (C-1″), 123.2 (C-6′), 123.1 (C-6″), 116.5 (C-5′, 5″), 115.2 (C-2′, 2″), 115.1 (C-8′), 115.0 (C-8″), 75.6 (C-1), 74.5 (C-4), 69.9 (C-3), 67.8 (C-5), 39.5 (C-2), 38.2 (C-6); ESI-MS *m*/*z* 517 [M + H]^+^. The above data were in agreement with those reported in the literature [35], so the structure was identified as isochlorogenic acid B.

Isochlorogenic acid A (**2**): light yellow powder; ^1^H-NMR (500 MHz, CD_3_OD) *δ* 7.61 (1H, d, *J* = 15.9 Hz, H-7′), 7.57 (1H, d, *J* = 16.0 Hz, H-7″), 7.07 (1H, d, *J* = 1.1 Hz, H-2′), 7.06 (1H, d, *J* = 1.1 Hz, H-2″), 6.97 (2H, dd, *J* = 8.2, 1.1 Hz, H-6′, 6″), 6.78 (2H, d, *J* = 8.2 Hz, H-5′, 5″), 6.37 (1H, d, *J* = 15.9 Hz, H-8′), 6.27 (1H, d, *J* = 16.0 Hz, H-8″), 5.45 (1H, m, H-5), 5.42 (1H, m, H-3), 3.94 (1H, dd, *J* = 8.4, 2.9 Hz, H-4), 2.30 (1H, m, H-6a), 2.18 (2H, m, H-2), 2.15 (1H, m, H-6b); ^13^C-NMR (125 MHz, CD_3_OD) *δ* 180.0 (C-7), 169.1 (C-9′), 168.6 (C-9″), 149.6 (C-4′), 149.5 (C-4″), 147.1 (C-3′), 147.0 (C-3″), 146.8 (C-7′), 146.8 (C-7″), 128.0 (C-1′), 127.9 (C-1″), 123.0 (C-6′, 6″), 116.6 (C-5′, 5″), 115.8 (C-2′), 115.4 (C-2″), 115.2 (C-8′, C-8″), 76.0 (C-1), 73.4 (C-3), 72.3 (C-5), 71.7 (C-4), 39.0 (C-6), 37.0 (C-2); ESI-MS *m*/*z* 517 [M + H]^+^. The above data were in agreement with the previous literature [35], so the structure was identified as isochlorogenic acid A.

### 3.4. Cell Culture and Electrophysiology

The procedure of cell culture and electrophysiology was performed according to literature [8]. In brief, the HEK293 cells were cultured at 37 °C under 5% CO_2_ incubation in Dulbecco’s minimal essential medium supplemented with 10% fetal bovine serum. For intracellular calcium measurement and whole-cell patch-clamp recording, HEK293 cells were seeded in a 12-well plate and on glass coverslips, respectively. HEK293 cells were transiently transfected with individual complementary DNA (cDNA) plasmids of human TRPV3. Isochlorogenic acids A and B (100 mM) and 2-APB (1 M) were dissolved in dimethyl sulfoxide (DMSO) and the final concentration of DMSO was less than 0.2%. The pipette solution and bath solution used for whole cell recording contained 130 mM NaCl, 3 mM *N*-2-hydroxyethylpiperazine-*N*-2-ethane sulfonic acid (HEPES), and 0.2 mm ethylene diamine tetraacetic acid (EDTA), and adjusted the pH value to 7.2. The current of TRPV3 channel was recorded in cell held at 0 mV in response to a voltage ramp from −100 to +100 mV for 500 milliseconds, and analyzed at ±80 mV. The data was processed by Origin 8.6 software (OriginLab, Northampton, MA, USA).

### 3.5. Molecular Docking

In order to develop insights into the nature of the binding interactions of isochlorogenic acids A and B, a molecular docking study was performed on human TRPV3 model in the present study [26]. The X-ray crystal structure of hTRPV3 (PDB code: 6MHO) was downloaded from the Protein Date Bank (http://www.rcsb.org) and prepared using AutoDockTools to keep one subunit from tetramer, and then the polar hydrogen atoms and Gasteiger charges were added to the macromolecular file. The 3D structures of compounds were depicted and minimized energy in Chem3D Ultra 8.0 (PerkinElmer, Waltham, MA, USA, version 12.0). The active pocket was enclosed in grid box (60 Å × 60 Å × 60 Å) with a certain grid spacing (0.375 Å), and the docking parameter file for ligand conformational searching was created using the Lamarckian genetic algorithm. Other parameters were set as default except for changing the docking runs to 100 replications [36]. The PyMOL (Schrödinger, New York, NY, USA) and LigPlot^+^ v.1.46 (European Bioinformatics Institute, Cambridge, UK) were used to generate all figures of structural models.

### 3.6. Selection of Extraction Solvent Systems for Sample Pretreatment

The ethyl acetate extract was conducted by a series of two-phase solvent systems as descibed in the literature with minor modification [32]. Ethyl acetate extract (4 mg) was dissolved in the lower phase (1 mL) of *n*-hexane-ethyl acetate-water (5:1:4, v/v/v), and then extracted three times with the upper phase (1 mL). The upper phase was drawn off and combined as Fr. I. The lower phase was collected and concentrated for the next two-phase solvent extraction. Using the same method, Fr. II and Fr. III were obtained in turn by using the two-phase solvent systems composed of *n*-hexane-ethyl acetate-water at the ratio of 3:3:4 and 1:5:4 (v/v/v). The fractions obtained from liquid-liquid microextraction were evaporated to dryness. The residues were dissolved in methanol and analyzed by HPLC.

### 3.7. Refinement of Crude Extract

In order to enrich the target compounds, the ethanol extract was refined by conventional solvent partition with single water-immiscible organic solvent and water and selected ternary solvent system. The ethanol extract (60 g) was suspended in water (800 mL) and successively extracted with *n*-hexane (600 mL × 3) and ethyl acetate (600 mL × 3), respectively. The ethyl acetate phase was combined and concentrated to produce dried product (2.6 g). Subsequently, the dried product was dissolved in the lower phase (650 mL) of *n*-hexane-ethyl acetate-water (3:3:4, v/v/v), and then extracted three times using equal volume of the upper phase. The two-phase solvent system *n*-hexane-ethyl acetate-water (1:5:4, v/v/v) was used to obtain Fr. III after the lower phase was concentrated. As a result, 1.7 g of Fr. III was obtained, which was used for following HSCCC preparation.

### 3.8. Selection and Preparation of Two-Phase Solvent System for HSCCC

A series of two-phase solvent systems, *n*-hexane-ethyl acetate-water with different ratios and in neutral and acidic conditions were further investigated using the conventional test tube method [37]. The *K* value was dependent on *A*_U_/*A*_L_, where *A*_U_ and *A*_L_ were recorded by the peak area of each target and nontarget compound in the organic phase and in the aqueous phase, following the results of HPLC analysis.

Then, *n*-hexane-ethyl acetate-water containing 1% acetic acid (1:4:8, v/v/v) was selected for the HSCCC separation. The desired volume of organic solvents and water containing 1% acetic acid was added into a separation funnel. The two phases were equilibrated thoroughly at room temperature. After separation, ultrasonic treatment was carried out for 0.5 h prior to use.

### 3.9. HSCCC Separation Procedure

The multilayer coiled column was first filled with the organic phase (stationary phase), and then the aqueous phase (mobile phase) was pumped into the column at a flow rate of 3.0 mL/min; meanwhile the apparatus at 25 °C was rotating at 900 rpm. Approximately 80 mg of the Fr. III was dissolved in 5 mL of the mobile phase. The sample solution was injected into the separation column, after the mobile phase was eluted from the tail outlet, and the system reached a hydrodynamic equilibrium. The effluent was immediately monitored with a UV detector at 254 nm and each fraction was manually collected based on the chromatogram.

### 3.10. HPLC Analysis

HPLC analyses were carried out on an Agilent 1260 system equipped with a YMC-Pack ODS-A column (250 × 4.6 mm, i.d., 5 μm) at 35 °C. The gradient elution mode with acetonitrile-water containing 2% acetic acid as follows: Acetonitrile: 0–10 min, 6%–6%; 10–20 min, 6%–10%; 20–45 min, 10%–20%; 45–80 min, 20%–35%. The effluent was monitored at 254 nm, and the flow rate was 1.0 mL/min.

## 4. Conclusions

A pair of natural caffeoylquinic acid derivatives (isochlorogenic acids A and B) were first isolated from the dried whole herbs of *A*. *alpina*, and identified as novel TRPV3 antagonists that both significantly attenuate TRPV3 current induced by agonist 2-APB. The putative binding sites of key residues His430 and Arg696 were predicted to mediate the open or closed state of hTRPV3 protein. To meet the demand of sufficient amounts of target compounds for application investigations, a comprehensive separation strategy was successfully established by HSCCC coupled with a liquid-liquid extraction approach in the present study. The result indicated that the present strategy is effective for the separation of isochlorogenic acids A and B using the two-phase solvent system composed of *n*-hexane-ethyl acetate-water containing 1% acetic acid (1:4:8, v/v/v), and the purities and yields of isolated compounds can be improved greatly. To the best of our knowledge, this is the first report of utilizing this comprehensive strategy for isolating isochlorogenic acid derivatives from the whole herbs of *A*. *alpina*. 

## Figures and Tables

**Table 1:** Partition coefficients (*K*) values of isochlorogenic acids A and B and the major nontarget impurity 10 in different two-phase solvent systems composed of *n*-hexane-ethyl acetate-water.

No.	Solvent System (*v*/*v*)	Partition Coefficient (*K*)			Separation Factor (*α*)	
		Isochlorogenic Acid B	Isochlorogenic Acid A	Impurity 10	Isochlorogenic Acids A and B	Isochlorogenic Acids B and Impurity 10
**1**	1:3:4	0.21	0.58	0.30	2.63	1.43
2	1:3:6	0.36	1.01	0.35	2.80	1.03
3	1:4:6	0.63	2.39	0.58	3.79	1.08
4	1:4:8	0.74	3.10	0.66	4.18	1.12
5	1:4:8 *	1.59	4.12	0.52	2.59	3.06

* Water containing 1% acetic acid.
